# What do oysters smell? Electrophysiological evidence that the bivalve osphradium is a chemosensory organ in the oyster, *Magallana gigas*

**DOI:** 10.1007/s00359-022-01608-4

**Published:** 2023-01-07

**Authors:** Ana Rato, Sandra Joaquim, Domitília Matias, Peter C. Hubbard

**Affiliations:** 1 Centre of Marine Sciences (CCMAR), University of Algarve, Campus de Gambelas, 8005-139 Faro, Portugal; 2 Department of Sea and Marine Resources, Portuguese Institute for Sea and Atmosphere (IPMA, I.P.), Av. 5 de Outubro s/n, 8700-305 Olhão, Portugal; 3 Interdisciplinary Centre of Marine Environmental Research (CIIMAR), University of Porto, Terminal de Cruzeiros do Porto de Leixões, Av. General Norton de Matos s/n, 4450-208 Matosinhos, Portugal

**Keywords:** EOsG, Electrophysiology, Chemoreception, Bivalve, Amino acids

## Abstract

The sensing of chemical cues is essential for several aspects of bivalve biology, such as the detection of food and pheromones. However, little is known about chemical communication systems in bivalves or the possible role of the osphradium as a chemosensory organ. To address this, we adapted an electrophysiological technique extensively used in vertebrates—the electro-olfactogram—to record from the osphradium in the Pacific oyster, *Magallana gigas*. This technique was validated using amino acids as stimulants. The osphradium proved to be sensitive to most proteinogenic l-amino acids tested, evoking tonic, negative, concentration-dependent ‘electro-osphradiogram’ (EOsG) voltage responses, with thresholds of detection in the range of 10^− 6^ to 10^− 5^ M. Conversely, it was insensitive to l-arginine and l-glutamic acid. The current study supports the hypothesis that the osphradium is, indeed, a chemosensory organ. The ‘electro-osphradiogram’ may prove to be a powerful tool in the isolation and characterization of pheromones and other important chemical cues in bivalve biology.

## Introduction

Most animals have evolved chemosensory systems, namely olfaction, in an adaptive way to detect and respond to chemical cues (Ache and Young 2005). In terrestrial animals, olfaction is considered as the chemical sense responsible for detecting volatile airborne chemicals, often at extremely low concentrations (Ache and Young 2005). In aquatic animals, such as fishes and crustaceans, chemoreception is restricted to water-soluble chemicals (Ache and Young 2005; Mollo et al. 2017). However, these organisms have chemosensory systems that, in many respects, are similar to those of terrestrial organisms (Ache and Young 2005).

In the marine environment, often with high turbidity and/or devoid of light, organisms depend heavily on these chemosensory systems to detect food, avoid predators and find conspecifics for reproduction (Emery 1992; Hara 1994; Derby and Sorensen 2008). The well-characterized olfactory system of fish, and antennules and other chemosensory systems of aquatic crustaceans, are known to be responsible. Much less is known about the chemosensory system of molluscs, particularly bivalves. In molluscs, chemoreception is thought to be mediated by the osphradium (Nezlin and Voronezhskaya 1997). However, whether the osphradium is a homologous organ in all extant molluscan classes is a matter of debate (Lindberg and Sigwart 2015).

The osphradium (or Spengel’s olfactory organ) was described by Spengel in 1881 as pigmented patches on the mantle epithelium of gastropods, bivalves and chitons, mostly likely representing olfactory sense organs (Lindberg and Sigwart 2015), whose main function was to test physicochemical properties of the water and, in many cases, to distinguish food properties (Kohn 1961).

This organ, located in the mantle cavity of the majority of molluscs, on or near the gills (Lindberg and Sigwart 2015), is typically innervated from the ctenidial nerve and composed of a sensory epithelium and ganglion connected, by the osphradial nerve, to the central nervous system (Nezlin and Voronezhskaya 1997; Lindberg and Sigwart 2015). In bivalves, these osphradia are either absent, in some species, or small, which makes them difficult to detect (Gosling 2004).

However, the definition and location of the osphradium within the phylum Mollusca are highly variable (see Haszprunar 1985a, b, 1987a, b; Lindberg and Sigwart 2015). In bivalves, the paired osphradium—pigmented or unpigmented—is located on or near the visceral ganglia, at the proximal part of the ctenidial nerve (Haszprunar 1987a; Lindberg and Sigwart 2015). Nevertheless, the exact function of the osphradium is still uncertain. It has been associated with regulation of cilia activity of gills on mussels (Aiello and Guideri 1964) or to function as a photoreceptor monitoring photoperiod to control behaviour and reproduction in oysters (Kraemer 1981). It was suggested by Haszprunar (1987a) that the osphradium has a role in spawning synchronization, being responsible for detecting chemical cues capable of inducing gamete release and therefore synchronize reproduction in broadcast spawner bivalve species (Haszprunar 1987a; Beninger et al. 1995; Lindberg and Sigwart 2015).

Although several studies address chemical communication and the role of the osphradium in gastropods and cephalopods (for example: Kohn 1961; Bailey and Laverack 1966; Emery 1992; Boal et al. 1999; Magel et al. 2007; Kamardin 2014; Kamardin et al. 2015; Simone 2021), little is known about chemical communication or chemosensory systems in bivalves. To overcome this lack, an electrophysiological technique widely used in vertebrates—the electro-olfactogram—was adapted to record from the osphradium in the Pacific oyster (*Magallana gigas*) and validated using amino acids as stimulants. The electro-olfactogram (EOG) is a direct current (DC) field potential recorded underwater, right above the olfactory epithelium and is believed to be the sum of the generator potentials of the olfactory receptor neurons as a response to a given odorant (Scott and Scott-Johnson 2002). To our knowledge, this was the first time an electrophysiological recording from the osphradium was successfully performed in bivalves.

## Materials and methods

### Animal preparation

Adult Pacific oysters (*Magallana gigas*, formerly *Crassostrea gigas*) (*n* = 15; 83.0 ± 8.4 g total weight and 9.2 ± 0.5 cm total length), collected from Ria de Alvor (South of Portugal, 37° 07′ 50″ N 8° 37′ 38″ W), were anaesthetized in aerated seawater containing 50 g L^− 1^ magnesium chloride (MgCl_2_) as suggested by Suquet et al. (2009). Oysters were considered anaesthetized when they gave no response to mechanical pressure on the valves. Once the adductor muscle had relaxed, the right valve was carefully removed by cutting the adductor muscle, without damaging the mantle, and a small incision was made in the gonads for sex identification. Oysters were then placed in natural aerated seawater and left overnight to recover and to clear the MgCl_2_ from the body, until use in EOsG recordings, usually on the following day, with successful recordings. Approval of ethics research is unnecessary in Portugal for work on non-cephalopod molluscs.

### Stimuli preparation

Immediately prior to use, glassware was rinsed with the same seawater used in stimuli preparation as suggested by Hubbard and Velez 2020. All twenty proteinogenic l-amino acids (Table 1) were selected as stimuli. Isomers of alanine (l-; d-; and β-alanine) and leucine (l- and d-leucine), the neurotransmitters serotonin (5-HT) and γ- Aminobutyric acid (GABA), and conspecific milt were also used as stimuli.

**Table 1:**
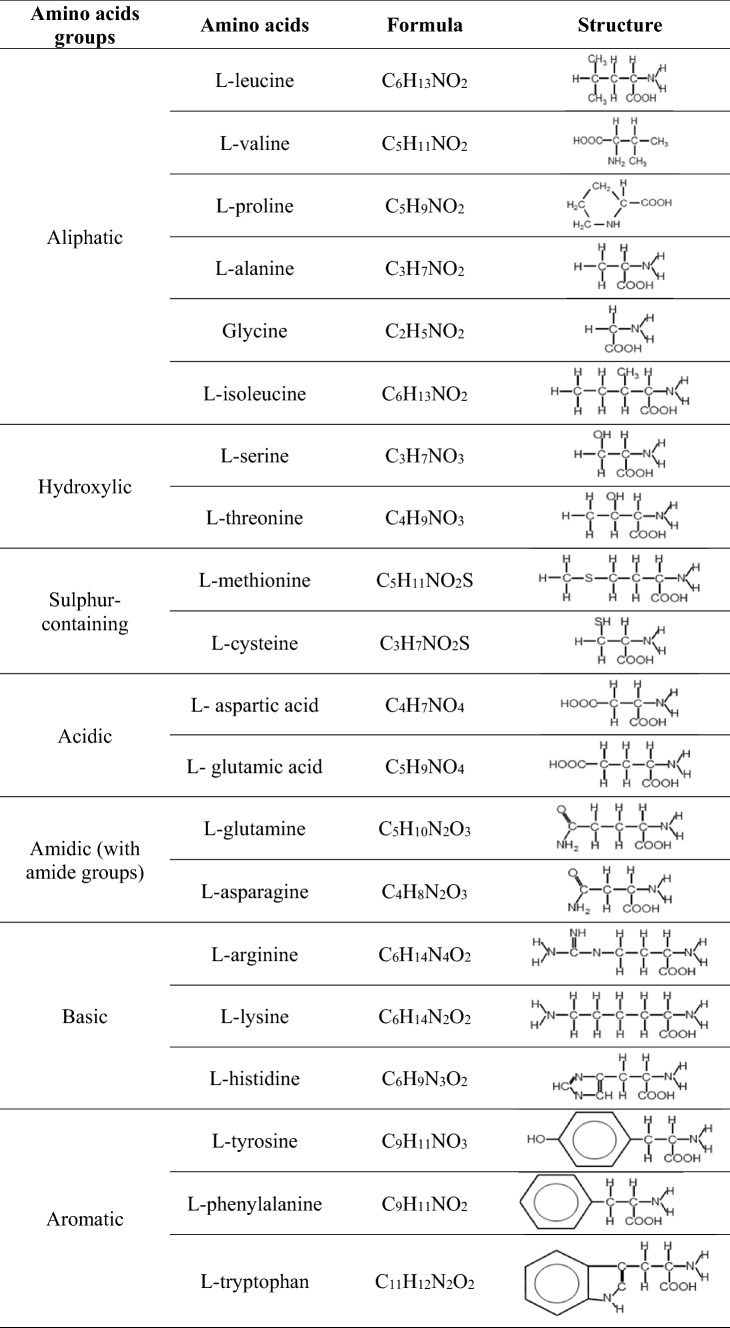
List of the amino acids group based on type of functional group, with the respective chemical formulas and schematic representation of the structures

Amino acids and neurotransmitter solutions were prepared by dissolving directly in charcoal-filtered natural seawater, at an initial concentration of 10^− 3^ M. Conspecific milt was obtained through natural spawning. Stimuli were prepared immediately before EOsG recordings.

l-Cysteine (10^− 3^ M) was used as positive control (standard), since it evoked strong and consistent responses in preliminary experiments, while negative control or blank was the water used to perform the dilutions but without any stimulus.

### Recording the electro-osphradiogram (EOsG)

The chemosensory activity of the osphradium was recorded through EOG, a well-established technique in our laboratory (Hubbard et al. 2002, 2011; Frade et al. 2002; Velez et al. 2005; Li et al. 2018).

**Fig. 1:**
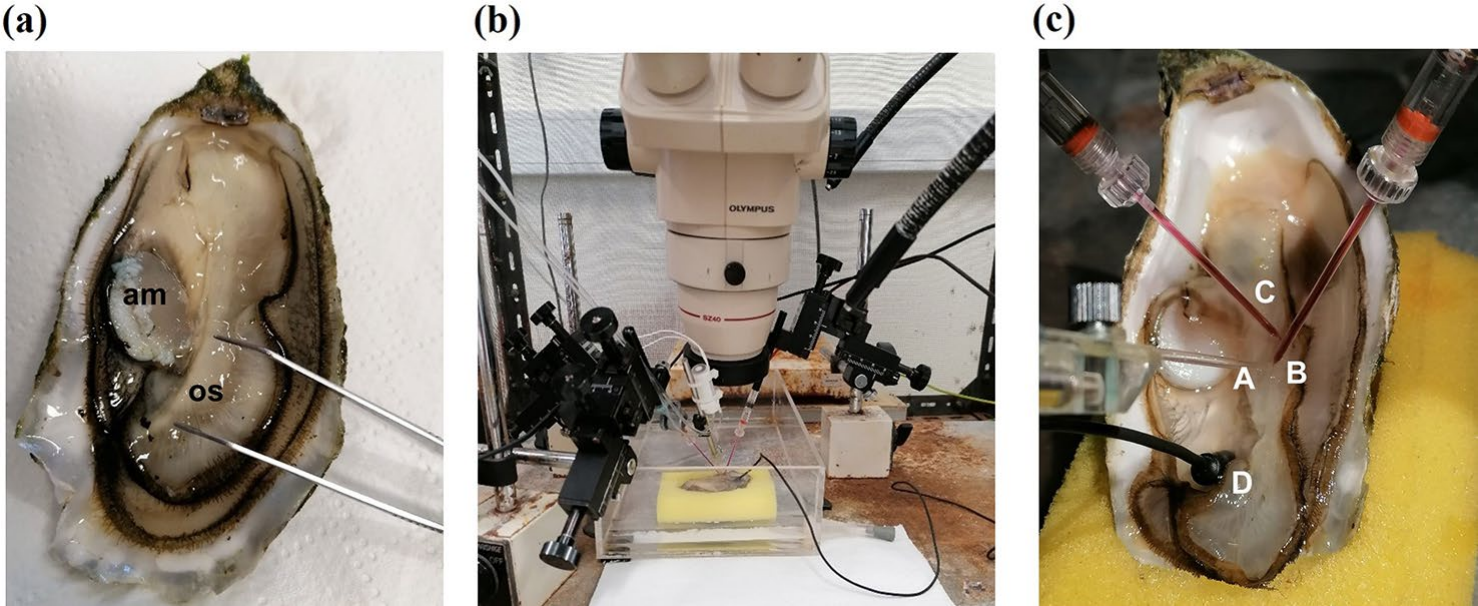
**a** Position of the osphradium (the tweezers indicate the location); *os* osphradium; *am* adductor muscle. **b** Electro-osphradiogram apparatus. **c** Placement of the electrodes and stimulus-delivery tube; (A) Stimulus-delivery tube; (B) recording electrode; (C) reference electrode; and (D) earth connection through silver/silver chloride pellet

The EOG equipment (Fig. 1), adapted to record from oysters, is composed of an experimental chamber, where the oysters were kept during the recordings without water, but being continuously irrigated with a flow of clean water.

The osphradium was under a continuous flow of clean, charcoal-filtered seawater, under gravity, via a glass tube, at a rate of 10 ml min^− 1^. Stimuli were introduced via a remotely operated solenoid valve, which allowed switching between control and stimulus solutions without interrupting the flow over the osphradium. The recording electrode (Fig. 1) was placed close to (but not touching) the osphradium, near the ventral area of the adductor muscle, and the reference electrode was placed close by on the mantle (not the osphradium) (Haszprunar 1987a). The optimal positions for electrodes and stimulus-delivery tube were determined using 10^− 3^ M l-cysteine as stimulus: the recording electrode was placed at a position that resulted in the largest response to 10^− 3^ M l-cysteine. The electrodes were made from borosilicate glass micropipettes, filled with 3 M NaCl in 4% agar and connected with the DC amplifier via Ag/AgCl pellets in 3 M KCl. Oysters were connected to earth via a silver/silver chloride pellet placed in the mantle cavity.

The voltage signal was amplified (✕2000 −✕20,000; Grass AC/DC strain gauge CP122, Astro-Med, West Warwick, Rhode Island, USA) with the low-pass filter set at 30 Hz. The signal was then digitized (Digidata 1440 A, Molecular Devices, Sunnyvale, California, USA) and recorded on a PC running Axoscope ^TM^ software (version 12.1, Molecular Devices).

Individual amino acids were given in order of increasing concentration (10^− 6^ M–10^− 3^ M), but the order of amino acids was varied among oysters. At least 1 min was allowed between successive stimuli. Throughout the recording period, blank and standard solution (10^− 3^ M l-cysteine) responses were recorded at regular intervals.

### Data treatment and statistical analysis

The peak amplitude was measured in millivolts. The amplitude of the response given to the blank was subtracted from all recorded responses, and these were then normalized to standard stimulus 10^− 3^ M l-cysteine similarly blank-subtracted.

Thresholds of detection were calculated by linear regression of the concentration–response curves of the log-transformed data, according to the formula log (*N* + 1.5) = *a.*log *C* + *b*, where *N* is the normalized response, *C* is the concentration, and *a* and *b* are constants. Therefore, the threshold of detection is the value for *x* where *y* = 0.1761 (i.e. log 1.5; *N* = 0).

Amino acids were grouped as described in Table 1, and concentration–response curves were plotted accordingly. Since the EOsG responses to concentrations of 10^− 6^ M (in the case of l-cysteine, l-serine, l-valine, l-histidine, l-threonine, l-lysine and l-asparagine) were close to zero and therefore similar to the blank, these concentrations were not considered for the calculation of thresholds of detection. One-way ANOVA followed by Tukey’s post hoc test was applied to determine statistical differences between isomers of the same amino acids, alanine and leucine. Since the current study aimed at a global vision and not an individual comparison, we chose standard error of the mean as a measure of data dispersion; therefore, results are expressed as mean ± standard error of the mean (SEM). The significance level was set at *P* ≤ 0.05. Statistical analysis was performed using software Sigmaplot (version 12.5).

## Results

**Fig. 2:**
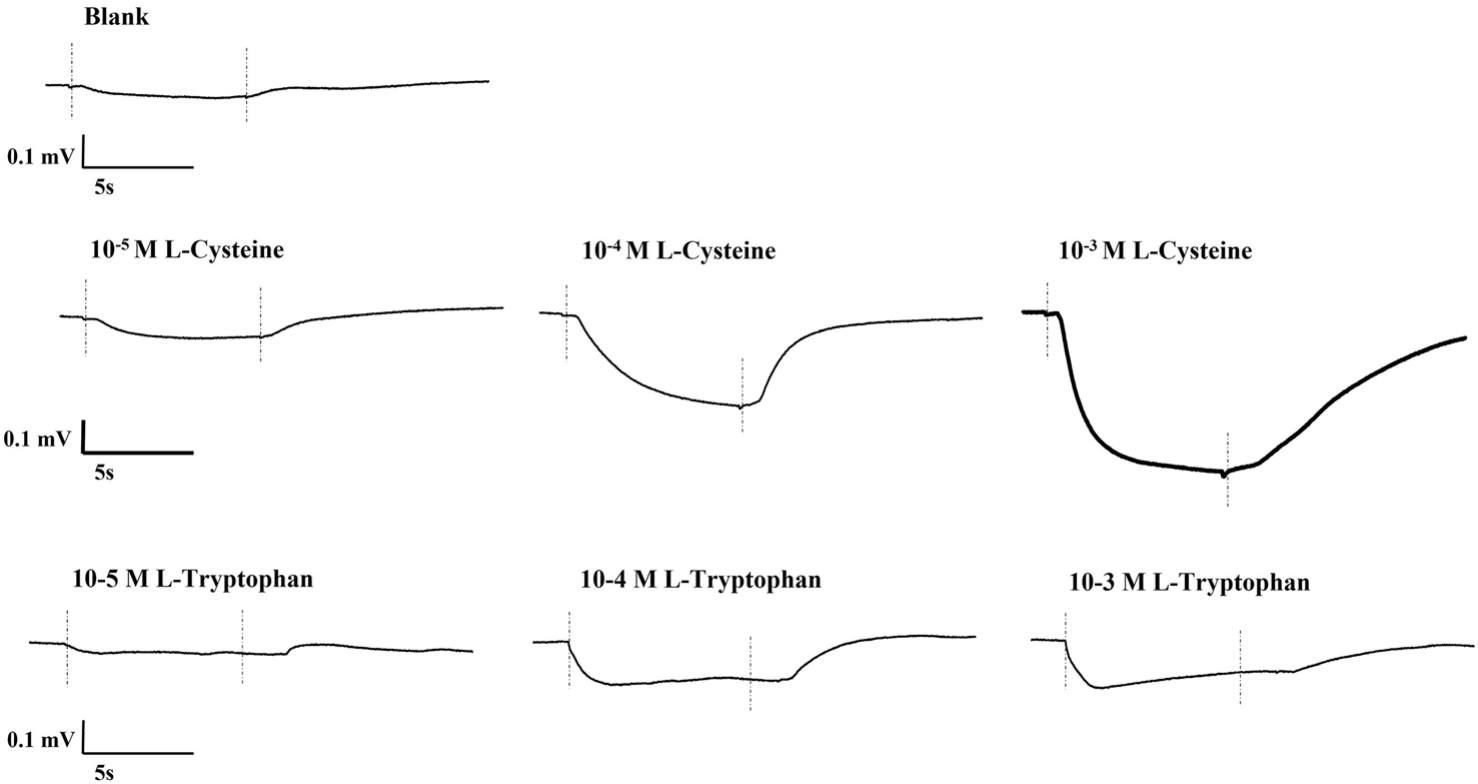
Typical EOsG responses to increasing concentrations (10^− 5^ M–10^− 3^ M) of l-cysteine and l-tryptophan. The dotted lines indicate the duration of stimulus delivery. A downward deflection of the trace is negative

The osphradium proved to be highly sensitive to most L-amino acids. The EOsG responses (Fig. 2) were characterized by a slow negative deflection at stimulus onset, followed by a tonic response during which the EOsG showed little or no sign of accommodation. When stimulus delivery ended, the potential returned to baseline levels within seconds. No clear differences were seen between sexes.

Figure 2 shows typical EOsG responses, at different concentrations (10^− 5^ M–10^− 3^ M), of l-cysteine and l-tryptophan, in comparison with the blank (seawater with no stimulus). In general, l-cysteine (10^− 3^ M) evoked stronger responses, with amplitudes up to − 0.92 mV, with an average amplitude of − 0.53 ± 0.09 mV, whereas responses to l-tryptophan (10^− 3^ M) were of lower amplitude, with a maximum and average amplitude of − 0.42 mV and − 0.24 ± 0.04 mV, respectively.

**Fig. 3:**
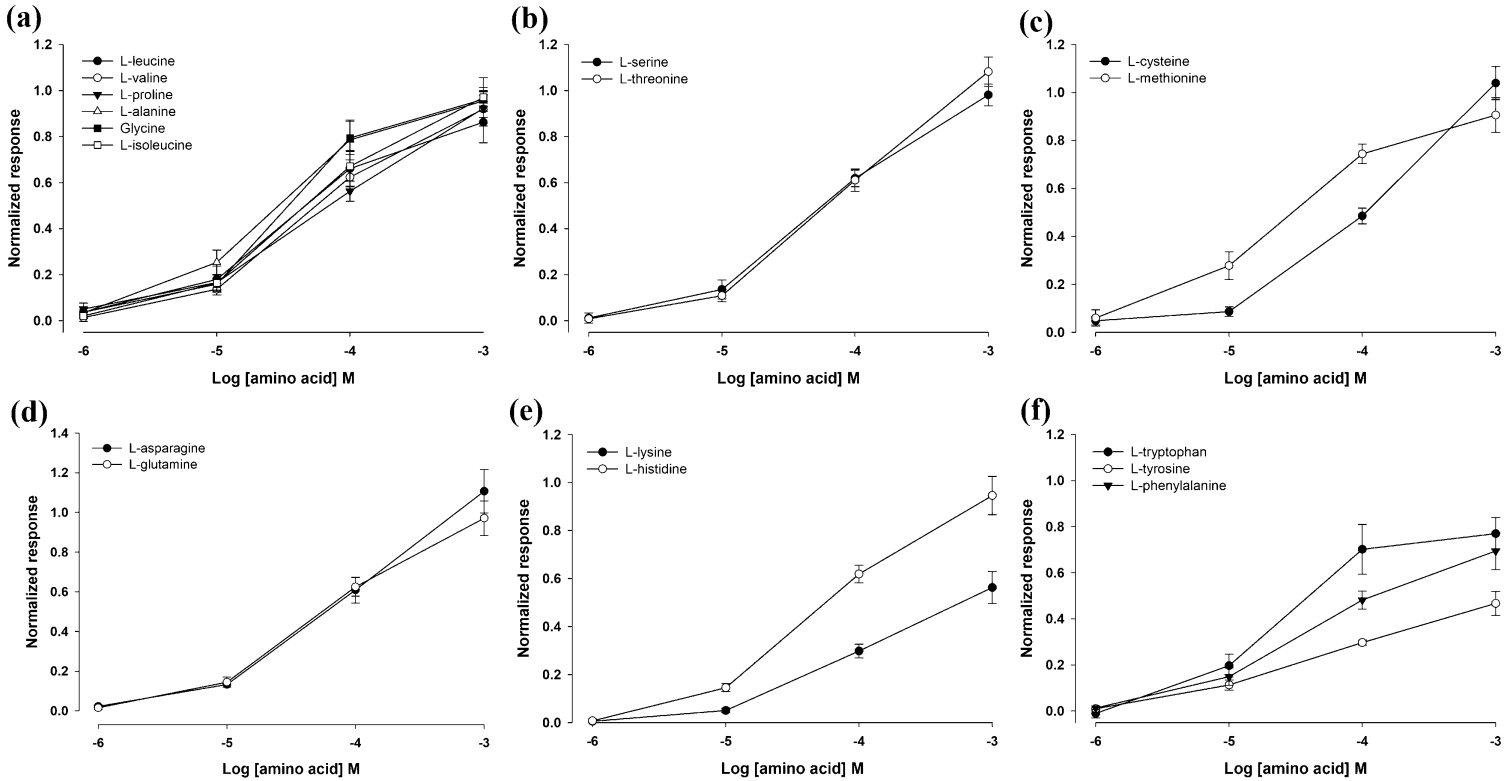
Semilogarithmic plot of the normalized electro-osphradiogram (EOsG) amplitude to amino acids: **a** aliphatic amino acids (*N* = 7; l-alanine: *N* = 5); **b** hydroxylic amino acids (*N* = 7); **c** sulphur-containing amino acids (*N* = 7); **d** amidic amino acids (*N* = 7); **e** basic amino acids (*N* = 7); **f** aromatic amino acids (*N* = 7) recorded from the osphradium. Data are shown as mean ± SEM

The amplitude of EOsG responses revealed to be strongly concentration-dependent (Fig. 3), and the EOsG response curves were similar in shape and amplitude within groups of amino acids. In general, aliphatic (Fig. 3a), hydroxylic (Fig. 3b), amidic (Fig. 3d) and sulphur-containing amino acids (Fig. 3c) evoked the strongest responses. In contrast, aromatic amino acids (Fig. 3f) elicited responses with half of the amplitude of the groups mentioned above, with normalized responses varying between 0.47 ± 0.05 and 0.77 ± 0.07 for l-tyrosine and l-tryptophan, respectively. Within this group, l-tryptophan showed a more sigmoidal curve, while l-phenylalanine and l-tyrosine showed more linear patterns.

In the group of sulphur-containing amino acids (Fig. 3c), l-cysteine exhibited a more linear pattern at higher concentrations (10^− 5^ M–10^− 3^ M), while l-methionine showed a more sigmoidal curve and lower normalized response at 10^− 3^ M (0.91 ± 0.07 vs. 1.04 ± 0.07 for l-cysteine).

**Fig. 4:**
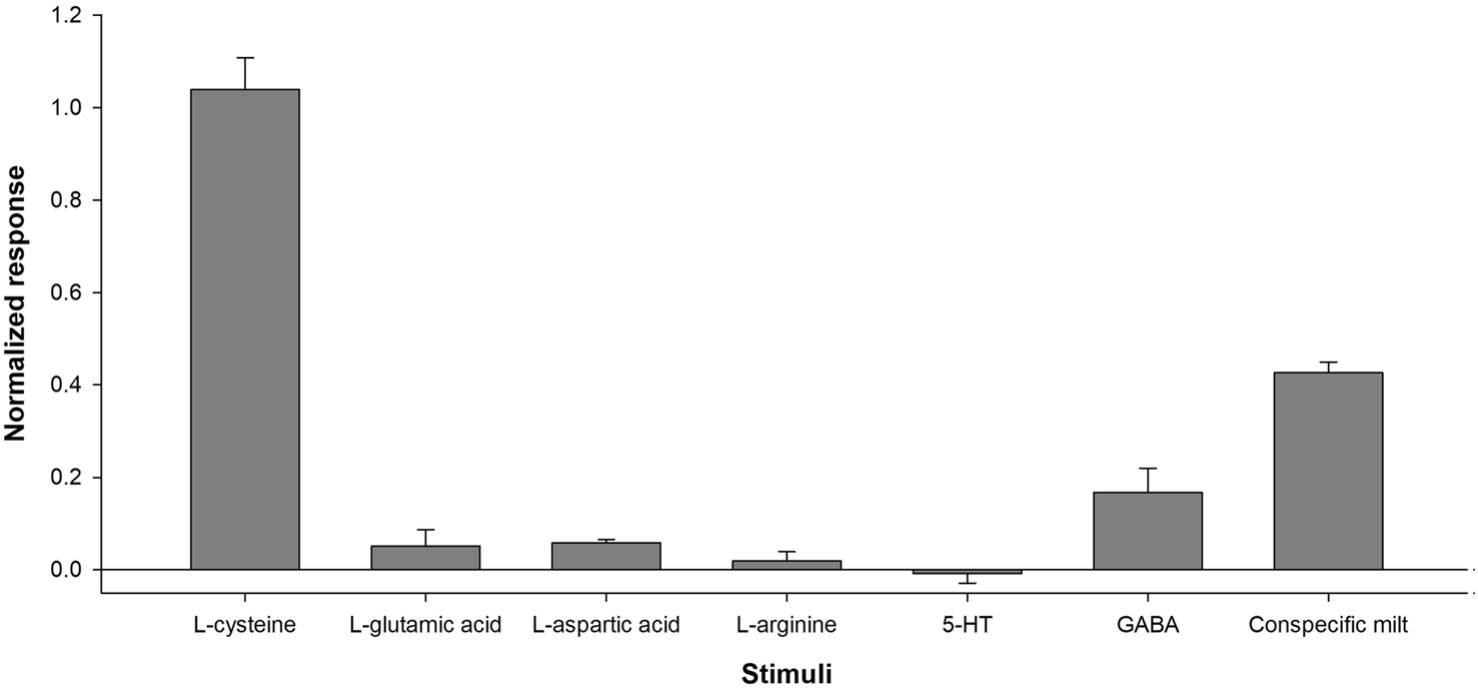
Normalized responses to compounds (10^− 3^ M) to which osphradium was less sensitive, compared to l-cysteine (*N* = 7). Amino acids: l-glutamic acid (*N* = 4), l-aspartic acid (*N* = 3), l-arginine (*N* = 4); neurotransmitter serotonin (5-HT) (*N* = 4) and γ-aminobutyric acid (GABA) (*N* = 3), and conspecific milt (*N* = 3). Data are shown as mean ± SEM

Within the basic amino acids (Fig. 3e), the evoked responses revealed to be highly variable, with L-arginine (Fig. 4) not being detected, even at the highest concentration tested (10^− 3^ M), l-lysine exhibiting a very low normalized response (0.56 ± 0.07) and L-histidine with a response similar in form and magnitude to that of aliphatic amino acids (0.95 ± 0.08).

On the other hand, acidic amino acids (l-glutamic acid and l-aspartic acid) did not evoke any response, even at higher concentrations (10^− 3^ M) (Fig. 4). l-Asparagine evoked the strongest response, exhibiting a normalized response of 1.11 ± 0.11, while the lowest normalized response was observed with l-tyrosine as stimulus (0.47 ± 0.05).

**Fig. 5:**
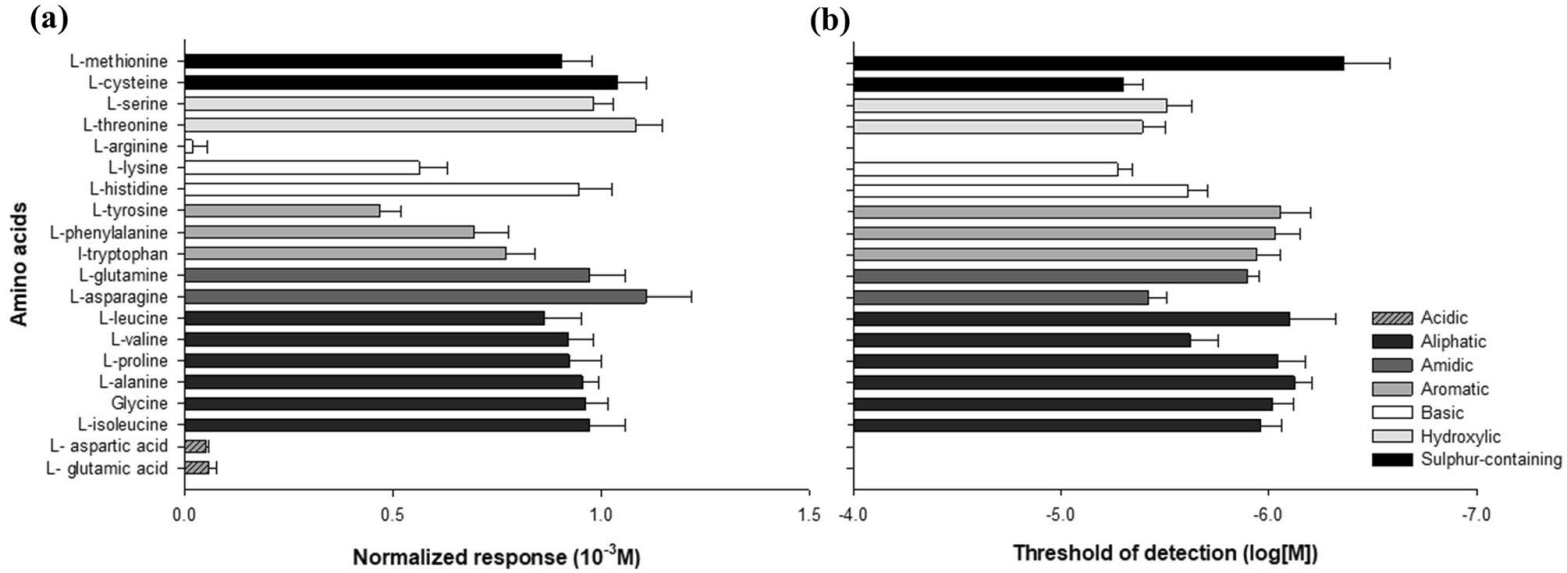
Osphradium sensitivity to the twenty proteinogenic amino acids. **a** Normalized responses (at 10^− 3^ M) and **b** calculated thresholds of detection of amino acids. Data are shown as mean ± SEM

The thresholds of detection (Table 2) varied between 10^− 6.36^ M and 10^− 5.27^ M for l-methionine and l-lysine, respectively. This suggests that, in most cases, concentrations below 10^− 6^ M are too low to be detected. The amino acids with the strongest response were not necessarily the most potent ones (Fig. 5). For instance, l-asparagine, which evoked the strongest normalized response (1.11 ± 0.11) revealed a threshold of detection of 10^− 5.42 ± 0.09^ M, while amino acids with weakest responses, as is the case of l-tyrosine and l-phenylalanine, exhibited lower thresholds of detection (10^− 6.06 ± 0.14^ M and 10^− 6.03 ± 0.12^ M, respectively), thus being more potent. l-methionine proved to be the most potent amino acid, with a threshold of detection of 10^− 6.36 ± 0.22^ M.

**Table 2:** Thresholds of detection for each amino acid

Amino acids	Thresholds of detection (M) ± SEM
l-Methionine	10^− 6.36 ± 0.22^
l-Alanine	10^− 6.12 ± 0.08^
l-Leucine	10^− 6.10 ± 0.22^
l-Tyrosine	10^− 6.06 ± 0.14^
l-Proline	10^− 6.04 ± 0.13^
l-Phenylalanine	10^− 6.03 ± 0.12^
Glycine	10^− 6.01 ± 0.11^
l-Isoleucine	10^− 5.96 ± 0.10^
l-Tryptophan	10^− 5.94 ± 0.12^
l-Glutamine	10^− 5.90 ± 0.06^
l-Valine	10^− 5.62 ± 0.13^
l-Histidine	10^− 5.61 ± 0.09^
l-Serine	10^− 5.51 ± 0.12^
l-Asparagine	10^− 5.42 ± 0.09^
l-Threonine	10^− 5.39 ± 0.11^
l-Cysteine	10^− 5.30 ± 0.09^
l-Lysine	10^− 5.27 ± 0.07^

Data are shown as mean ± SEM

**Fig. 6:**
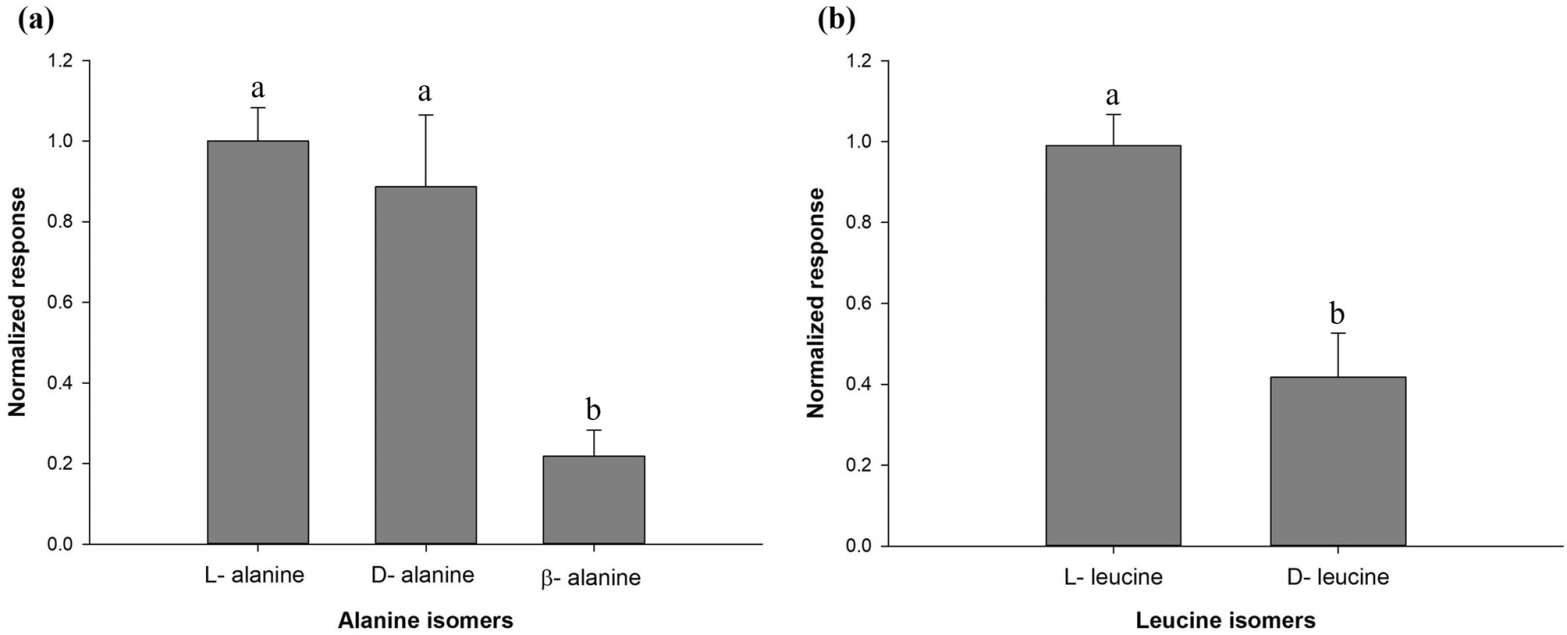
Osphradium sensitivity to different isomers. Normalized EOsG responses to different isomers of **a** alanine (L, D, β) and **b** leucine (L, D). Data are shown as mean ± SEM. Different letters indicate significant differences (ANOVA followed by post hoc Tukey test, *P* < 0.05)

Different isomers of alanine (L, D and β) and leucine (L and D) evoked different amplitude EOsG responses at the same concentration (Fig. 6). β-Alanine evoked significantly lower response (ANOVA, *F* = 12.54, *df* = 2, *P* = 0.001) than l- and d-isomers, whereas l-leucine evoked significantly larger responses (ANOVA, *F* = 18.35, *df* = 1, *P* = 0.003) than its d-isomer.

In addition to amino acids, oysters were also exposed to serotonin, GABA and conspecific milt (Fig. 4). Serotonin did not evoke any EOsG response, while GABA evoked an extremely low response, around 0.2 of normalized response. Conspecific milt evoked a strong EOsG response similar in form and magnitude to those evoked by amino acids.

## Discussion

As in the olfactory system of fishes (Caprio 1978; Hara 1994; Kasumyan 2004), the bivalve osphradium proved to be sensitive to amino acids, albeit with slightly higher thresholds of detection. The EOsG responses in oysters are distinct from those in vertebrates, being slower and of lower amplitude than those of fishes and lacking the fast-adapting initial phasic response. For example, in the chameleon cichlid (*Australoheros facetus*), a freshwater fish, the standard stimulus l-serine (10^− 5^ M) evoked EOG amplitudes about 4.5 times higher (3.77 ± 1.38 mV) (Hubbard et al. 2017) than those evoked by l-serine (10^− 3^ M) (0.85 ± 0.208 mV) in oysters. However, if one compares the responses evoked by oysters (0.53 ± 0.09 mV) to those evoked by saltwater fishes, as is the case of Senegalese sole (*Solea senegalensis*) (Velez et al. 2005), the responses evoked by the standard stimulus (l-cysteine, 10^− 3^ M) are similar in amplitude, around 0.5 mV for sole and 0.53 ± 0.09 mV in oysters. This is due to the fact that EOG carried out in seawater is less sensitive than that in freshwater, as a result of the water conductivity—the higher the conductivity, the lower the resistance and therefore the amplitude will be lower (Hubbard et al. 2011; Hubbard and Velez 2020). Given that the electro-osphradiogram is a DC voltage signal measured in seawater, the method may slightly underestimate the true sensitivity, as has been shown in marine fish (Hubbard et al. 2011).

In vertebrates, the EOG is characterized by a phasic response with a rapid negative deflection at the beginning of stimulus exposure, followed by an adaption period and a slower-adapting tonic response (e.g. Chaput 2000; Frade et al. 2002; Hubbard et al. 2003b, 2011; Lalloué et al. 2003; Velez et al. 2005; Eom et al. 2009; Lapid and Hummel 2013), whereas in oysters, the EOsG was characterized by a slow negative deflection at stimulus onset, followed by a tonic response with little or no sign of accommodation. The return to baseline levels occur within seconds. This difference may be because oysters are sessile organisms, and the only decision to make in a presence of a certain odorant is to open or close the valves, rather than actively follow an odour plume (Atema 2012), such as mobile animals.

The electrode position within the osphradium, as well as the variability between individuals, may have caused some fluctuation in the recorded responses for the same amino acid. This variation highlights the importance of normalizing the recorded responses to the standard stimulus, in this case, l-cysteine (10^− 3^ M).

The use of magnesium chloride (MgCl_2_), a muscle relaxant widely used as anaesthetic in bivalves (Culloty and Mulcahy 1992; Butt et al. 2008; Suquet et al. 2009; Alipia et al. 2014), was efficient to prevent the contraction of the adductor muscle and therefore keep the shell open (Butt et al. 2008; Suquet et al. 2009; Azizan et al. 2021). However, MgCl_2_ blocks calcium channels in the membrane of presynaptic terminals (Namba et al. 1995; Azizan et al. 2021) and consequently interferes with electrical signal transduction. Indeed, there was little or no response to amino acids immediately after exposure to MgCl_2_ (data not shown). However, after overnight recovery in clean seawater, it was possible to record from the osphradium; oysters are able to recover within 24 h with no physiological effects caused by MgCl_2_ (Namba et al. 1995).

Like fishes, oysters were highly sensitive to aliphatic (e.g. l-leucine, l-valine, glycine), hydroxylic (e.g. l-serine and l-threonine), amidic (l-asparagine and l-glutamine), and to sulphur-containing amino acids such as l-cysteine and l-methionine (Velez et al. 2005; Hubbard et al. 2011). Apparently, and in contrast to fishes, oysters are more selective in which amino acids they detect. The rank order of potency in oysters also differs from that described for fishes. For instance, in blackspot seabream (*Pagellus bogaraveo*) and in Senegalese sole (*Solea senegalensis*), l-proline proved to be the least potent amino acid (Velez et al. 2005; Hubbard et al. 2011), whereas, in oysters, it was highly potent. l-Arginine, which evoked a strong response in the upper epithelium of Senegalese sole as well as is pointed as one of the most stimulative amino acids for goldfish (Rolen et al. 2003; Velez et al. 2005), did not evoke any response in oysters (Fig. 4). Neither did l-glutamate nor l-aspartic acid (Fig. 4) evoke any response in oysters. This suggests that, like marine fishes, oysters are less responsive to acidic amino acids (l-glutamic acid and l-aspartic acid) (Velez et al. 2005; Hubbard et al. 2011). However, in the marine gastropod *Buccinum undatum*, these acidic amino acids were the most effective chemical stimuli, inducing strong responses even at low concentrations (Bailey and Laverack 1966).

The apparent sensitivity to amino acids was lower than that in fishes, with thresholds of detection between 10^− 6.36^ to 10^− 5.27^ M, while in fishes, like Mozambique tilapia (*Oreochromis mossambicus*) and other teleosts, and blackspot seabream, the threshold of detection ranged from 10^− 9^ to 10^− 5^ M (Kasumyan 2004; Hubbard et al. 2011; Kutsyna et al. 2016). Moreover, Kutsyna et al. (2016) observed that amino acids with lower thresholds of detection elicited higher EOG amplitudes, in the Mozambique tilapia. The same pattern was seen in oysters. For example, l-asparagine and l-cysteine evoked larger amplitude EOG responses and the thresholds of detection were correspondingly lower. However, some amino acids, such as l-tyrosine and l-phenylalanine, evoked lower EOG amplitudes but had relatively low thresholds of detection (Fig. 5).

Similar to fishes, oysters seem to be more responsive to l-amino acids, probably due to their ubiquity in nature and their involvement in food identification and location (Hara 1994; Velez et al. 2007). Amino acids are potent odorants for aquatic organisms, inducing strong responses and triggering feeding behaviour in a wide variety of species, such as fishes (e.g. Hara 2006), crustaceans (e.g. Fuzessery and Childress 1975), gastropods (e.g. Bailey and Laverack 1966; Croll 1983; Wedemeyer and Schild 1995; Magel et al. 2007), larval amphibians (Arzt et al. 1986; Heerema et al. 2018) and, in the current study, bivalves. The fact that several groups of invertebrates (e.g. gastropods and crustaceans) detect amino acids may indicate that amino acid chemoreceptors may be a common feature among invertebrates (Bailey and Laverack 1966). However, due to the use of slightly different experimental approaches, it is not possible to directly compare oysters with other invertebrates.

As in fishes (Hubbard et al. 2003a), the osphradium of oysters proved to be highly sensitive to conspecific milt. In fact, in spawning trials, besides physical stimulation (e.g. thermal shock), conspecific sperm is widely used as an additional stimulus to induce oysters, namely females, to spawn. Since oysters are external fertilizing broadcast spawners, it is crucial for conspecifics to be able to detect the gametes released in order to synchronize spawning and therefore maximize fertilization. This may suggest a role of the osphradium in such spawning synchronization, as proposed by Haszprunar (1987a). However, further research is needed including, but not limited to, identifying the chemicals involved.

The neurotransmitter serotonin is known to act as a spawning inducer in bivalves (Gibbons and Castagna 1984), while γ-aminobutyric acid (GABA) is known for its role as an inducer of settlement and metamorphosis in bivalve larvae (García-Lavandeira et al. 2005; Mesías-Gansbiller et al. 2008, 2013). That these two compounds did not evoke any EOsG response in oysters may suggest a direct effect on the reproductive and/or nervous system of bivalves, rather than via the osphradium. Thus, although serotonin is known as a spawning inducer in bivalves (Gibbons and Castagna 1984), it cannot be considered a pheromone.

The knowledge of the chemosensory role of the osphradium in bivalves may be relevant in the development of aquaculture techniques. Recently, there has been an increasing demand for alternative diets for bivalves (Knauer and Southgate 1996; McCausland et al. 1999; Arney et al. 2015; Rato et al. 2018). This technique may prove useful in the formulation of alternative diets, by seeking to include, in the formulation, food-related odours with higher olfactory potency and therefore improve the overall acceptance by bivalves.

To our knowledge, this was the first time an EOsG recording was successfully carried out in any bivalve and strongly supports the hypothesis that the osphradium is a chemosensory organ (Haszprunar 1987a) in this taxon, as it is in other molluscs. Subsequently, a whole series of questions about chemoreception in bivalves may finally be answered. How do bivalves perceive the surrounding environment? What is the role of chemical cues in reproduction and predator avoidance? Are they able to detect predators or conspecifics nearby? The ‘electro-osphradiogram’ (EOsG) may prove to be a powerful tool in the isolation and characterization of pheromones and other important chemical cues for bivalves. Future research on bivalve chemoreception, as well as establishing how widely applicable the ‘electro-osphradiogram’ is to other bivalves, is needed to fully understand the role of the osphradium as a chemosensory organ.
